# A Noise Based Medical Elites Silence Model and Public Health Opinion Distortion in Social Networks

**DOI:** 10.3389/fpubh.2021.791893

**Published:** 2022-01-14

**Authors:** Jianliang Wei, Chi Qin, Hao Ji, Lingling Guo, Jingjing Chen, Yingying Xu

**Affiliations:** ^1^School of Management Engineering and E-Commerce, Zhejiang Gongshang University, Hangzhou, China; ^2^School of Environment and Resource, Zhejiang Agriculture and Forestry University, Hangzhou, China; ^3^College of Chemical Engineering, Zhejiang University of Technology, Hangzhou, China; ^4^Zhijiang College, Zhejiang University of Technology, Hangzhou, China; ^5^School of Economics and Management, University of Science and Technology Beijing, Beijing, China

**Keywords:** misinformation, opinion propagation, information noise, social network, public health, distortion

## Abstract

Under the impact of internet populism, internet violence, and other noises on the internet, medical elites, who have a professional background, did not intend to share their opinions on the internet. Thus, misinformation about health is increasingly prevalent. We roughly divided the users in social networks into ordinary users, medical elites, and super-influencers. In this paper, we propose a communication model of health information based on the improved Hegselmann-Krause (H-K) model. By conducting MATLAB-based simulation, the experimental results showed that network noise was an important factor that interfered with opinion propagation regarding health. The louder the noise is, the harder it is for health opinions within a group to reach a consensus. But even in a noisy environment, super-influencers could influence the overall cognition on public health in the social network fundamentally. When the super-influencers held positive opinions in public health, the medical elite keeping silent had a noise-tolerant effect on opinion communication in public health, and vice versa. Thus, three factors concerning noise control, the free information release of medical elites, and the positive position of super-influence are very important to form a virtuous information environment for public health.

## Introduction

In recent years, social media platforms (e.g., Facebook, Twitter, and WeChat^1^) have been widely adopted for information communication. Facebook had 2.8 billion monthly active users by the end of 2020, and its daily active users reached 1.8 billion (1). There are thousands of updates per second. Compared with traditional media such as newspapers, radio, and television, these online social media tools are changing the way information is shared and opinions exchanged. However, for public health, low-credibility information is increasingly prevalent in social networks. Thus, the dissemination of misinformation has become an important factor affecting the cognition of public health concepts. The misinformation not only prevents individuals from engaging in behaviors, but also likely raises public health risks, and even prevents the public from considering policy choices regarding health issues. In the field of vaccinations alone, the World Health Organization declared that measles cases had gone up 300% in 2019 because of the absence of vaccinations.

The prevalent social network platforms have greatly prompted the spread of misinformation about public health and brought about a series of deviant propagation effects. Firstly, the elderly surge into social network platforms amid aging societies. Due to their lesser capacity to screen information online and low sensitivity to health-related information, the elderly tend to be misguided. Secondly, some self-publishing and social media exaggerate or even fabricate their health-related posts as stunts or for profitable purposes. These highly controversial and contagious contents are prone to the formation of information cascades (2, 3). Thirdly, as the prevalence of cyber-gangs and network violence rise, influential medical elites are more inclined to keep silent, which narrows the space for accurate and authoritative health information to spread (4). All these facts significantly aggravate the prevalence of misinformation about public health, leading to distorted choices from individuals.

In this work, we proposed a revised H-K model, which adopts a new coefficient named noise to reflect the consequence of medical elite silence to health information opinion exchange in social networks. Based on the traditional opinion leaders influence theory (5), we divided users into three categories including ordinary users, super-influencers, and elite users. Medical elites refer to opinion leaders in the medical field, and super-influencers are taken as super opinion leaders. Base on this, we designed parameters for the targeted opinion interaction model according to the different attributes of users, and proposed the misinformation (about health) propagation model based on medical elites, and conducted the simulation. This paper may the first concern of elite silence in social networks using a quantitative method, and help to understand the mechanism of how distortion in health opinion propaganda happened.

The rest of the paper is organized as follows. Section Related Literature is the review of related literature. The concept of network noise is put forward in section Proposed Approach, and the opinion exchange model focus on the silence of medical elites silence is constructed. In section Simulation and Discussions, five groups of experiments based on Matlab are conducted, and results are discussed. Conclusions are in the final section.

## Related Literature

At present, the dissemination of misinformation about public health in social networks has attracted wide attention. In this section, we reviewed the literature regarding misinformation about public health, opinion distortion, and propagation.

### Misinformation in Social Networks

In the public health field, social media, unfortunately, is playing a negative role in spreading misinformation, especially for immunization and infectious diseases. During the spread of the Ebola virus, Pathak et al. (6) found plentiful misleading information about Ebola online, and a large part of the misinformation comes from opinion leaders who are highly active on social networks, which caused rumors to be prevalent over evidence-based information. Tustin et al. (7) reported the widespread misinformation about side effects, as well as mistrust in government or pharmaceutical companies, on vaccination. From the perspective of chronic non-infectious diseases, Chua and Banerjee (8) reported that online users are more likely to trust and share cancer-related misinformation if the misinformation is pessimistic instead of optimistic. While dealing with urological health information, Loeb et al. (9) found that a significant amount of circulating information on social platforms is commercial, biased, or misinformative.

### Opinion Distortion in Social Networks

Jafari and Navidi (10) described the relationship between users using “edge” parameters, which differ among the four attributes: directional chain, bi-directional chain, follower and successor, and friendship state. They quantified the reasons for the public opinion evolution and opinion deviation. Li et al. (11) integrated the non-trust relationships between users, which could trigger opinion distortion online as a core parameter into the evaluation model. Based on real data, Hosseinipozveh et al. (12) found that non-trusting relationships widely existed in social networks where opinions could spread without barriers and finally lead to opinion distortion. Katz et al. (13) believed that interpersonal relationships play an important role in information spreading through the public and assessed the extent of its influence through empirical analysis. Also, information itself influences distortion. Later, Chadwick et al. (14) studied four datasets based on a customized survey of individual blogs, interactive websites, news articles, and Twitter users during the 2017 British election campaign, and found that an important factor causing democratic misinformation and disinformation behavior is the sharing of “tabloid news.” Lu et al. (15) quantitatively estimated the distorted opinions of users by mining controversial topics in social networks and found that opinions would change over time. Tucker et al. (16) also believed that political polarization was closely related to online disinformation, and the use of social media positively promoted such a relationship. The study also found that individuals' distortion of information content would eventually evolve into a collective distortion of opinion. Wei and Meng (17) address online opinion distortion from a super-influencer perspective and put forward a simulation model to explain the phenomenon. Sasahara et al. (18) focus on the unfriending effect on opinion exchange and find that very small changes of influence and unfriending can cause online communities to become segregated.

### User Role in Social Network

The differentiation of user roles is the key to studying the evolution and dissemination of network opinions. Previously, Valente et al. (5) simulated the path of opinion propagation and proved that opinion leaders could speed up information propagation. Clauset et al. (19) found that independent spreaders in a scale-free network were closely related to the occurrence of public opinion deviation. This study believed that such special nodes, which are unaffected by their neighbors, could spread information through ways other than the links, and change the attitudes of non-neighbors, causing cross-range deviation of public opinion values. Li et al. (20) proposed the Ising model and studied the mismatch and the interactions among nodes within social networks, and found that nodes with high degrees tended to be connected by nodes with low degrees, which took the super-influencers as the benchmark for reference and imitation and thus led to the opinion distortion. In terms of elite users, based on their influence and information transmission mode, Anagnostopoulos et al. (21) defined elite users as bloggers, celebrities, media organizations, and representatives of other official organizations. Khan (22) defined elite users as “people who have extremely disproportionate access to or control over a resource.” Based on a real-time data analysis, Bastos et al. (23) found that only 10–15% of users retweeted the posts from traditional media and more generally of original views and high-quality content from elite users.

To sum up, although some studies focused on the influences of special nodes on public opinion propagation, and a few recent pieces of literature had mentioned super-influencers (2, 24), the vast majority ignored the influence of elite users and their silence effect; even Claerhoudt (25) who expressed concern over the influence of noise from neighbor to opinion leader, did not study the silence effect. Although elite users do not possess the super-power the super-influencers do, they are producers of high-quality information and spokespeople of original opinions (26), especially in the field of public health. Facing increasingly complex social networks, medical elites have gradually become the “silent users.” It is urgent to discuss and analyze the mechanism of silent elites affecting the dissemination of health misinformation and how they could influence the overall cognition of health misinformation in social networks.

## Proposed Approach

In social networks, social status and authority differ among individuals, which to some extent influences the opinion interactions among individuals. Chen et al. (27) distinguished opinion leaders from ordinary users by four aspects: reputation, stubbornness, attraction, and polarity. Further, they studied the problem of information propagation when two opinion leaders held opposing opinions on a social network. It is believed that elites or opinion leaders are not as easily able to change their point of view as ordinary users. Further, Cheng et al. (28) argued that the extent of confidence and the initial opinion value were the primary characteristics to distinguish opinion leaders from ordinary users. The initial opinion values of opinion leaders were close to 0 or 1 and had a high degree of confidence. According to the degree of confidence, opinion leaders could be further divided into super-influencers and general opinion leaders. The initial opinions of super-influencers who had the highest level of confidence were distributed in the interval (0, 0.25]⋃[0.75, 1). While the general opinion leaders who were less confident had initial opinion values distributed in the interval (0.25, 0.75). Relatively, the ordinary users had a minimum opinion value.

Therefore, combined with the characteristics of the health field, we defined medical elites as select medical professionals that are superior in terms of ability or qualities to the rest, and extracted the following features of medical elites:

- Opinions were stable. Considering the seriousness of the field of public health, elite users were not so easy to be influenced by their neighbors, especially those who were ordinary users.
- Opinions were influential. Due to their professionality, opinions of medical elites were easy to spread with less of a limitation threshold.
- Opinions were sensitive. Medical elites had less time to participate in discussion online because of their occupation, which had led to a phenomenon of “silence” within the noisy online world.

At the same time, there were a few rather influential super-influencers in the field of public health, which is defined as few users who have tremendous influences not only in social network platforms, but also in the real world; they have many followers, their released information always triggers much attention and discussion, they are super leaders who's influence can cross domains, fans (17). For example, during the Coronavirus epidemic, opinions from authoritative experts like Prof. Zhong Nanshan and Prof. Zhang Wenhong were more persuasive than those of other medical elites. Their opinions radiate strongly and have an overturning impact on the public. In this study, both the super-influencers and medical elites were taken into consideration while constructing the propagation model of misinformation about public health.

### Improved Hegselmann-Krause Model

The H-K model is based on the bounded confidence principle. There we considered a group with size *N*, each node within it held an initial opinion on a topic which was represented by *x*_*i*_(0), and *x*_*i*_ was a random value in the continuous interval[0, 1], where 0 indicated that the node held an extremely negative opinion and 1 indicated that the node held an extremely positive opinion. At any moment, we randomly chose a node *i* from the group. When there was another node *j* whose opinion had differed from that of node *i* not more than a threshold ε, i.e., |*x*_*i*_ − *x*_*j*_| ≤ ε, node *j* was added into *N*_*i*_—the neighbor set of node *i*. We specifically described it as *N*_*i*_(*t*) = {*j* ∈ {1, 2, ⋯ , *N*}:|*x*_*i*_ − *x*_*j*_| ≤ ε}. When node *i* had screened and filtered all the members in the system, it would take the neighbor set as the opinion interaction set at the next moment. Then, the weighted average value of the opinions of all the nodes in this set would become the new opinion value of node *i*. The opinion interactions could be described by the equation:$x_{i}\left(t+1\right)=\overset{n}{\underset{j=1}{\sum }}w_{ij}\left(t\right)x_{j}\left(t\right)$, where *w*_*ij*_ represented the weight of influence that node *j* exerted on node *i*, and satisfied $\overset{n}{\underset{j=1}{\sum }}w_{ij}=1$.

The H-K model supports that nodes that hold different opinions exert the same extent of influence on each other. While in the health field, people are inclined to accept opinions that are more similar than their own, and the credibility of the opinion holders matters a lot. In order to accurately simulate the opinion interaction of health information, we made the following improvements to the H-K model:

**Definition 1:** we denoted the set of opinions of all nodes within social network G as *X*(*t*) = {*x*_1_(*t*), *x*_2_(*t*), ⋯ , *x*_*n*_(*t*)}, where *x*_*i*_(*t*) was the opinion value of node *i* at time *t*, and satisfied *x*_*i*_(*t*) ∈ [0, 1]. Particularly, *x*_*i*_(0) was the initial opinion of node *i*. The opinion values depended on the users, there we defined 0 as the extreme negative health information and 1 as the extreme positive health information.

**Definition 2:** we denoted the opinion neighbor set of node *i* at time *t*, and satisfied *N*_*i*_(*t*) = {*j* ∈ {1, 2, ⋯ , *N*}:|*x*_*i*_ − *x*_*j*_| ≤ ε}.

**Definition 3:** we denoted the confidence matrix as a non-negative matrix*C* of size *N* × *N*, where *C*_*ij*_ was the confidence level of node *i* to node *j*, *i, j* ∈ *G*, when *i* = *j*, *C*_*ij*_ denoted the confidence level of node *i*. And *C*_*ij*_ ∈ {0.1, 0.2, ⋯ , 0.9}, the confidence level increased with the increase of *C*_*ij*_.

**Definition 4:** we set *u*_*i*_ as the influence coefficient of node *i*.

**Definition 5:** we set *f*_*ij*_ as the influence of node *i* on node *j* which increased with the increase of *f*_*ij*_, and satisfied the formula: $f_{ij}=\left[1/\max\left(|x_{i}\left(t\right)-x_{j}\left(t\right)|,\sigma \right)\right]\cdot \left[C_{ij}C_{ji}/d_{ij}^{2}\right]$. Where *d*_*ij*_ was the distance between node *i* and node *j*, and σ was an infinitely-small positive real number which ensured that the formula made sense when the difference between opinion values of node *i* and *j* was 0. Particularly, when the opinion values of node *i* and *j* were infinitely close to or even equal, node *i* had a huge influence on node *j*.

**Definition 6:** we set ε*C*_*ij*_ as the opinion interaction threshold of node *i* on *j*. Node *i* interacted with node *j* on opinions if and only if |*x*_*i*_ − *x*_*j*_| ≤ ε*C*_*ij*_ where the confidence coefficient ε ∈ [0, 1].

**Definition 7:** we set *w*_*ij*_(*t*) as the weight of influence that node *i* assigned to node *j* at time *t*. When |*x*_*i*_ − *x*_*j*_| ≤ ε*C*_*ij*_, there was *w*_*ij*_(*t*) = *u*_*j*_*f*_*ij*_, otherwise, *w*_*ij*_(*t*) = 0.

### Considerate Medical Elites

Based on the opinion evolution rule of the improved H-K model, when there were only elite users and ordinary users in social network, the opinion interactions between nodes satisfied the following algorithm:


(1) $$\{\begin{array}{c} x_{i}\left(t+1\right)=\sum_{j\in N_{i}}\frac{w_{ij}\left(t\right)}{\sum_{j\in N_{i}}w_{ij}\left(t\right)}x_{j}\left(t\right)\\ x_{j}\left(t+1\right)=\sum_{i\in N_{j}}\frac{w_{ij}\left(t\right)}{\sum_{i\in N_{j}}w_{ij}\left(t\right)}x_{i}\left(t\right) \end{array}$$

The opinion interaction formula above shared the same idea with the H-K model that a node accepted the opinions of neighbors with different weights when it decided to update its opinion the next time. The difference was that we differentiated the influence weight of different types of nodes according to the characteristics that each node had a different influence and trust degree. There were four cases of opinion-update during the interactions among different types of nodes:

(1) When there was no elite user *e* within the neighbor set of an ordinary user *i*, i.e., the node *j* that interacted with node *i* must be an ordinary user, and thus the interaction condition satisfied |*x*_*i*_ − *x*_*j*_| ≤ ε*C*_*ij*_;
(2) When there was an elite user *e* within the neighbor set of an ordinary user, the interaction between ordinary user *i* and elite user *e* satisfied |*x*_*i*_ − *x*_*e*_| ≤ ε*C*_*ie*_, otherwise the same as (1);
(3) When there were only ordinary users *j* adjacent to the elite user *e*, the interaction between the ordinary user *j* and elite user *e* satisfied |*x*_*j*_ − *x*_*e*_| ≤ ε*C*_*je*_;
(4) When elite users were neighbors, the interaction between elite users *e*_1_ and *e*_2_ satisfied |*x*_*e*_1__ − *x*_*e*_2__| ≤ ε*C*_*e*_1_*e*_2__.

### Considerate Super-Influencer

Based on the introduction of elite users, we further introduced the super-influencers of health information. Within the interactions among different types of nodes, there were five situations of updating opinions of health cognition:

(1) When there was neither any elite user *e* nor super-influencer *s*, i.e., the node *j* that interacted with node *i* must be an ordinary user, and thus the interaction condition satisfied |*x*_*i*_ − *x*_*j*_| ≤ ε*C*_*ij*_;
(2) When there existed both a super-influencer and an elite user, the interaction between ordinary user *i* and elite user *e* satisfied |*x*_*i*_ − *x*_*e*_| ≤ ε*C*_*ie*_, and the interaction between ordinary user *i* and super-influencer *s* would not be limited by the threshold, and interaction conditions among ordinary users were the same as (1);
(3) When there were only ordinary users *j* adjacent to the elite user *e*, the interaction between the ordinary user *j* and elite user *e* satisfied |*x*_*j*_ − *x*_*e*_| ≤ ε*C*_*je*_;
(4) The interaction between elite user *e*_1_ and *e*_2_ satisfied |*x*_*e*_1__ − *x*_*e*_2__| ≤ ε*C*_*e*_1_*e*_2__, the interaction between elite user *e*_1_ and super-influencer *s* would not be limited by the threshold;
(5) The super-influencer would not be interfered with by threshold and would not change their original opinion while interacting with others, no matter what types of users were adjacent to the super-influencer *s*.

### Medical Elite Silence Model Based on Noise

In the complex public opinion environment, with the growing absence of opinions expressed by elite users, the informal opinions and the emotional expressions led by “covenant-lite” and “anonymity” have caused the unhealthy development of the public opinion ecosystem. Here, we defined violent behaviors on the internet, mean language, slander, privacy violation and etc., as the main forms of network noise, it is a degree to reflect the unfriendly language and behavioral environment toward a person, an entity, or society. Network noise will make medical elites with expertise and a higher social status become hesitate to release information, comment selectively, and finally, to be silent. Thus, network users' health cognition was distorted consequently.

Therefore, this paper argued that while studying the propagation of health information online, we should not only consider the interactive modes and restrictions between individuals but also pay more attention to the behavioral changes of nodes with different identities, especially the elite silence, under the interference of network noise. In this paper, we simulated the network noise using Gaussian white noise, and to study the influence of elite users on health information propagation due to the reduction of speech frequency when they were disturbed by noise. So, we made the following improvements to the opinion interaction conditions and the opinion updating algorithm in the model:

(1) When there was neither any elite user nor super-influencer adjacent to node *i*, i.e., any node that involved in interactions must be an ordinary user*j*, and the interaction condition satisfied |*x*_*i*_ − *x*_*j*_| ≤ ε*C*_*ij*_. Thus, based on the improved H-K model, the algorithm of opinion interactions among ordinary users should include the noise factor, that was:
  (2) $$\{\begin{array}{c} x_{i}\left(t+1\right)=\sum_{j\in N_{i}}\frac{w_{ij}\left(t\right)}{\sum_{j\in N_{i}}w_{ij}\left(t\right)}x_{j}\left(t\right)+I\alpha \left(t\right)\\ x_{j}\left(t+1\right)=\sum_{i\in N_{j}}\frac{w_{ij}\left(t\right)}{\sum_{i\in N_{j}}w_{ij}\left(t\right)}x_{i}\left(t\right)+I\alpha \left(t\right) \end{array}$$
  where α(*t*) was the extent to which ordinary user *i* was affected by network noise and uniformly distributed on the interval[0, 1]; *I*represented the intensity of network noise that received by an ordinary user *i*, *I* ∈ [0, 1].
(2) When there were unusual users adjacent to the ordinary user *i*, then the interaction between the ordinary user *i* and the elite user *e* satisfied |*x*_*i*_ − *x*_*e*_| ≤ *IεC*_*ie*_; and the interaction between the ordinary user *i* and the super-influencer *s* would not be limited by the threshold.
(3) When there were only ordinary users *j* adjacent to the elite user *e*, the interaction between the ordinary user *j* and elite user *e* satisfied |*x*_*e*_ − *x*_*j*_| ≤ *IεC*_*ej*_, and the opinion updating algorithm was $x_{e}\left(t+1\right)=\underset{j\in N_{e}}{\sum }\frac{w_{ej}\left(t\right)}{\underset{j\in N_{e}}{\sum }w_{ej}\left(t\right)}x_{j}\left(t\right)+I\alpha \left(t\right)$.
(4) When there were authoritative users adjacent to the elite user *e*_1_, the interaction between elite users *e*_1_ and *e*_2_ satisfied |*x*_*e*_1__ − *x*_*e*_2__| ≤ ε*C*_*e*_1_*e*_2__; the interaction between the elite user *e*_1_ and the super-influencer *s* would not be limited by the threshold, the opinion updating algorithm was the same as above.
(5) The super-influencer would not be interfered with by the threshold and change their original opinion while interacting with others, no matter what types of users that adjacent to the super-influencer were.

It was worth noting that, in the environment with network noise, when|*x*_*e*_ − *x*_*i*_| ≤ *IεC*_*ei*_, the influence weight of elite users *w*_*ei*_(*t*) = *u*_*e*_*f*_*ei*_, otherwise,. At the same time, because of network noise, individual opinion values *x*_*i*_(*t*) might exceed the interval[0, 1] in the process of opinion evolution. Thus, in order to guarantee the valid range of opinion values, the termination condition of opinion evolution was considered, i.e., $\overset{n}{\underset{i=1}{\sum }}\left[x_{i}\left(t+1\right)-x_{i}\left(t\right)\right]\leq \delta$, where was the termination threshold of opinion evolution and was a positive number that approaches 0 indefinitely.

## Simulation and Discussions

### Experiment Design

In this paper, we proposed a scale-free network based on degree value non-probabilistic adding-edge algorithm, then simulated and deduced the evolutionary process of health opinion using MATLAB. In the experiment, we performed simulation with a population of 1,000 nodes, and set the node with the highest degree as the super-influencer, the number of medical elites was limited to 5–10% of the population, and the rest were the ordinary users. At the same time, we let the initial opinions of nodes in the network be uniformly distributed in the interval[0, 1]. We thought that the medical elites were more positive so their opinions were set within the interval[0.65, 0.75]. As for the super-influencer, its initial value of negative opinion was 0.2, and the positive one was 0.95. Meanwhile, the basic opinion evolution ran 500 iterations, and the interaction threshold among users ε = 0.5. The opinion convergence is reached when the disparity of opinions within the group ≤ 0.01.

### Simulation and Discussion

#### Simple Simulation of Public Health Opinion Evolution

**Figure 1:**
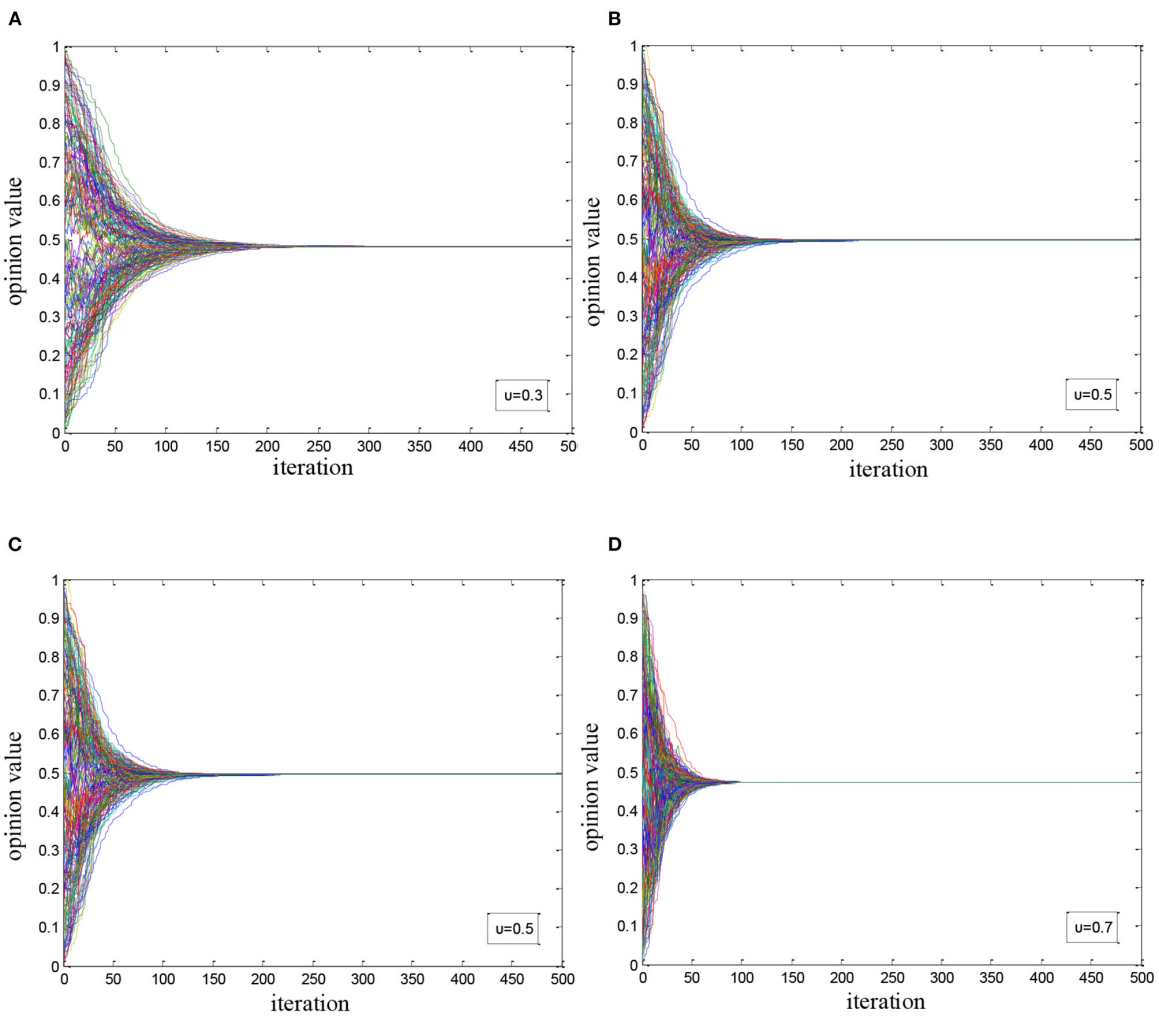
Simulation of health opinion evolution under pure environment: **(A)** *u* = 0.1; **(B)** *u* = 0.3; **(C)** *u* = 0.5; and **(D)** *u* = 0.7.

Considering the situation that there were only ordinary users, we changed the influence coefficient *u* among them and simulated the evolutionary process of health opinion, and take the results as the control group to the following experiment group. In the experiment, we gradually increased *u* from 0.1 to 0.9 constantly and recorded the condition of public opinion evolution when *u* equaled to 0.1, 0.3, 0.5, and 0.7, respectively. As shown in Figure 1, it is found that in an ideal network environment, the convergence value of health opinions is 0.5, and the number of iterations needed for health opinions to transform the condition from divergence to convergence is determined by the influence coefficient among users, and they are also negatively correlated.

#### With Medical Elites and Ordinary Users Involved

**Figure 2:**
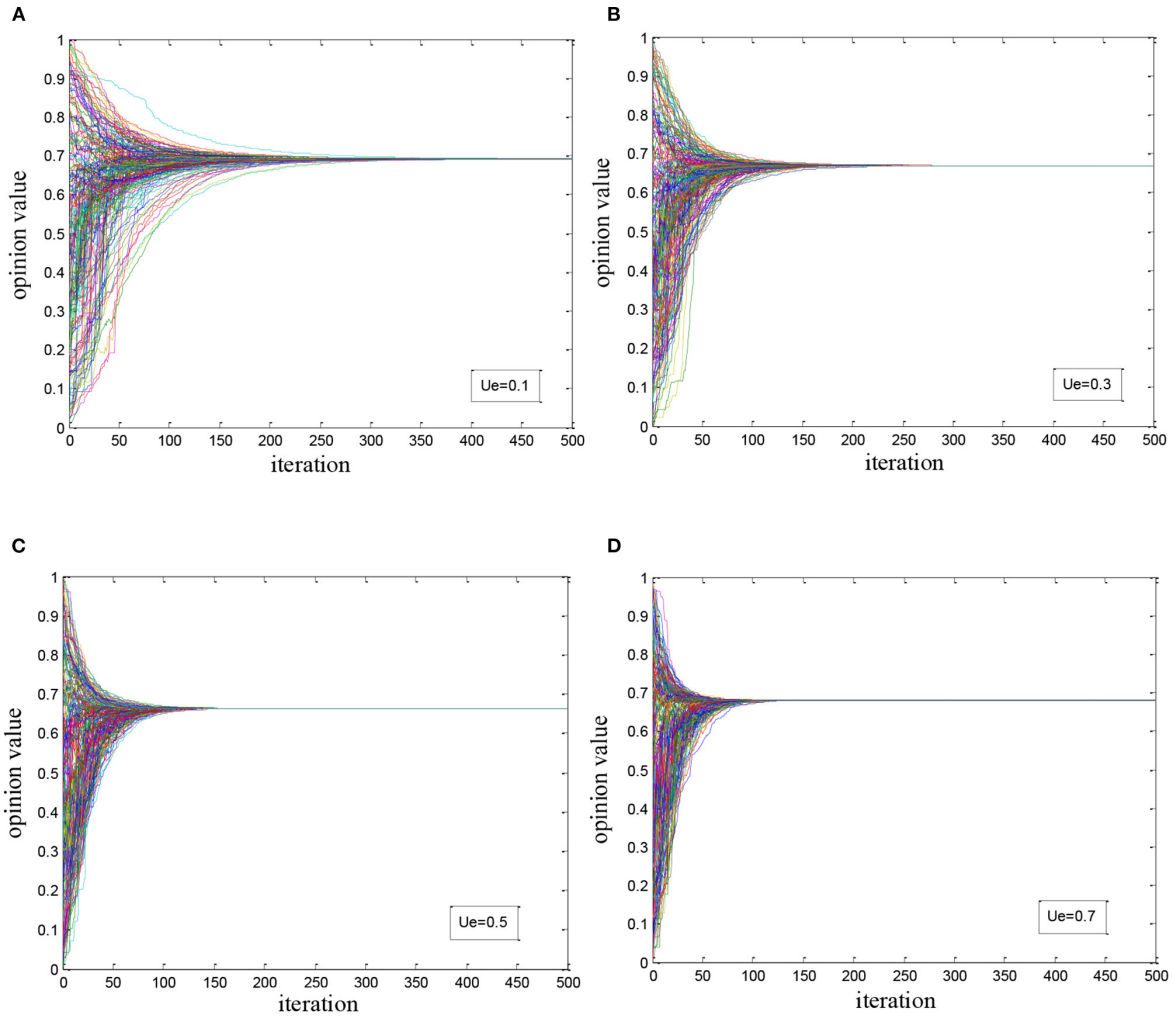
Simulation of health opinion evolution with elites and ordinary users coexisting: **(A)** *u*_*e*_ = 0.1; **(B)** *u*_*e*_ = 0.3; **(C)** *u*_*e*_=0.5; **(D)** *u*_*e*_ = 0.7.

We introduced the medical elites based on the experiment of simple simulation, and the remaining constraints were kept unchanged. The number of medical elites and their initial opinions satisfied the setting in the experimental design. We tried to observe the evolution of health opinions by debugging the influence coefficient of elite users *u*_*e*_. In the experiment, we observed the trend of network opinion evolution when equaled to 0.1, 0.3, 0.5, and 0.7, respectively. As shown in Figure 2, it is found that:

(1) The overall health opinion converges at approximately 0.7, which is conformed to the initial opinions of medical elites. We could state that the overall health opinion gradually reaches a consensus led by medical elites, and the consensus opinion conforms to the initial opinions of medical elites.
(2) With the increase of the influence coefficient of medical elites *u*_*e*_, the convergence rate of health opinions within-group increases accordingly. As shown in the experiment, the number of iterations to reach a consensus reduces from 300 to 100.

#### With Super-Influencer Involved

**Figure 3:**
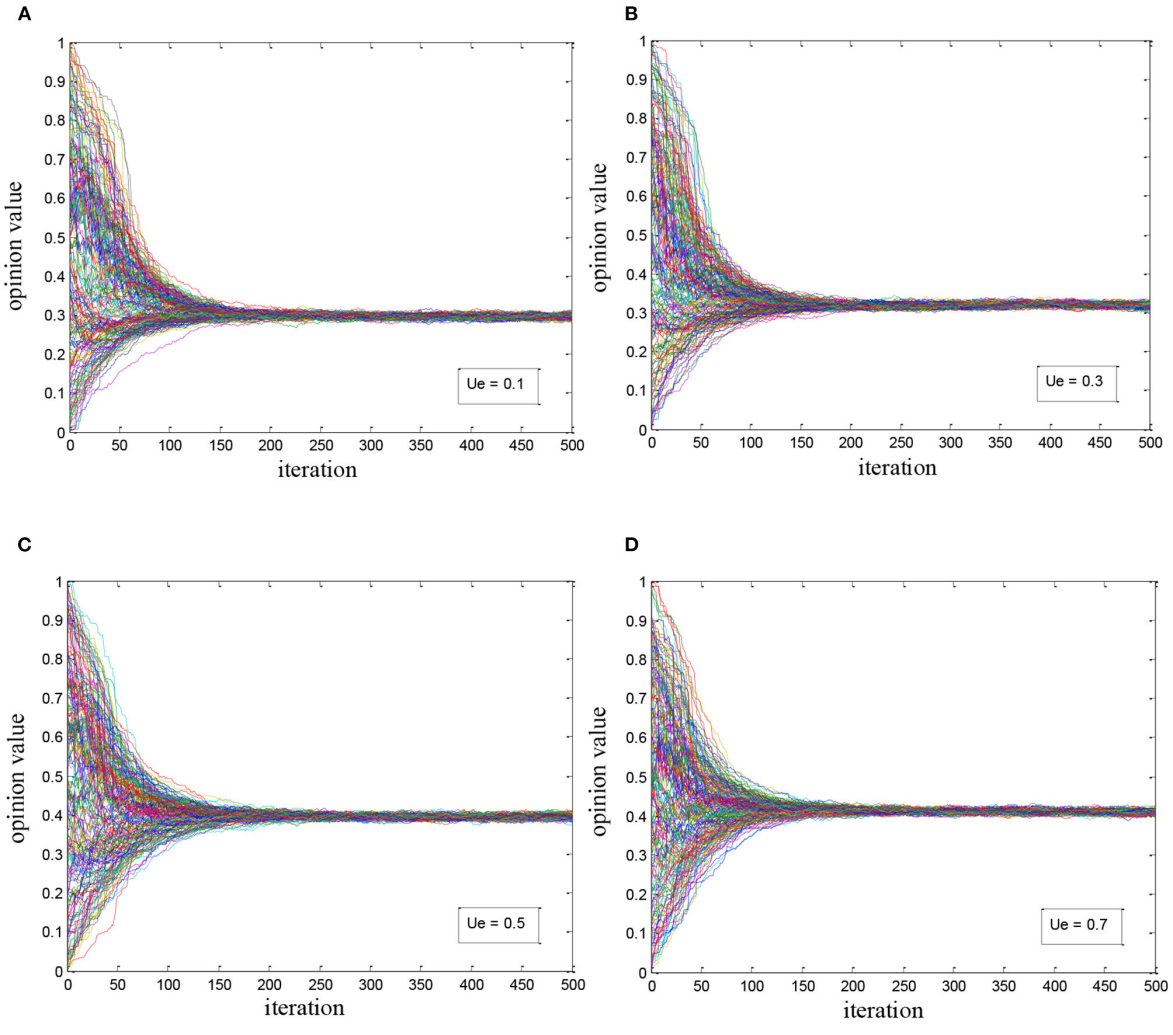
Health opinion evolution considering the super-influencer: **(A)** *u*_*e*_ = 0.1; **(B)** *u*_*e*_ = 0.3; **(C)** *u*_*e*_ = 0.5; **(D)** *u*_*e*_ = 0.7.

Based on the second experiment, we introduced the super-influencer with an initial value of 0.25 which represented the negative health opinion. We tried to observe the evolution of health opinions by debugging the influence coefficient of medical elites *u*_*e*_, and made the coefficient *u*_*e*_ equal 0.1, 0.3, 0.5, and 0.7, respectively as the watchpoints in the experiment. The results are shown in Figure 3. It is found that:

(1) Influenced by the super-influencer, the overall health opinion converges to the interval [0.3, 0.45] which is closer to the opinion of super-influencer but not totally dominated by it.
(2) Medical elites have guided the overall health opinion to some extent. With the coefficient *u*_*e*_increasing, the overall health opinion and its convergence value are gradually moved “away from” the opinion value of super-influencer. When *u*_*e*_ reaches 0.5, the convergence value of the overall health opinion increases by 0.1, showing the important role of authority as the opinion influence of medical elites.

#### Considerate Network Noise

**Figure 4:**
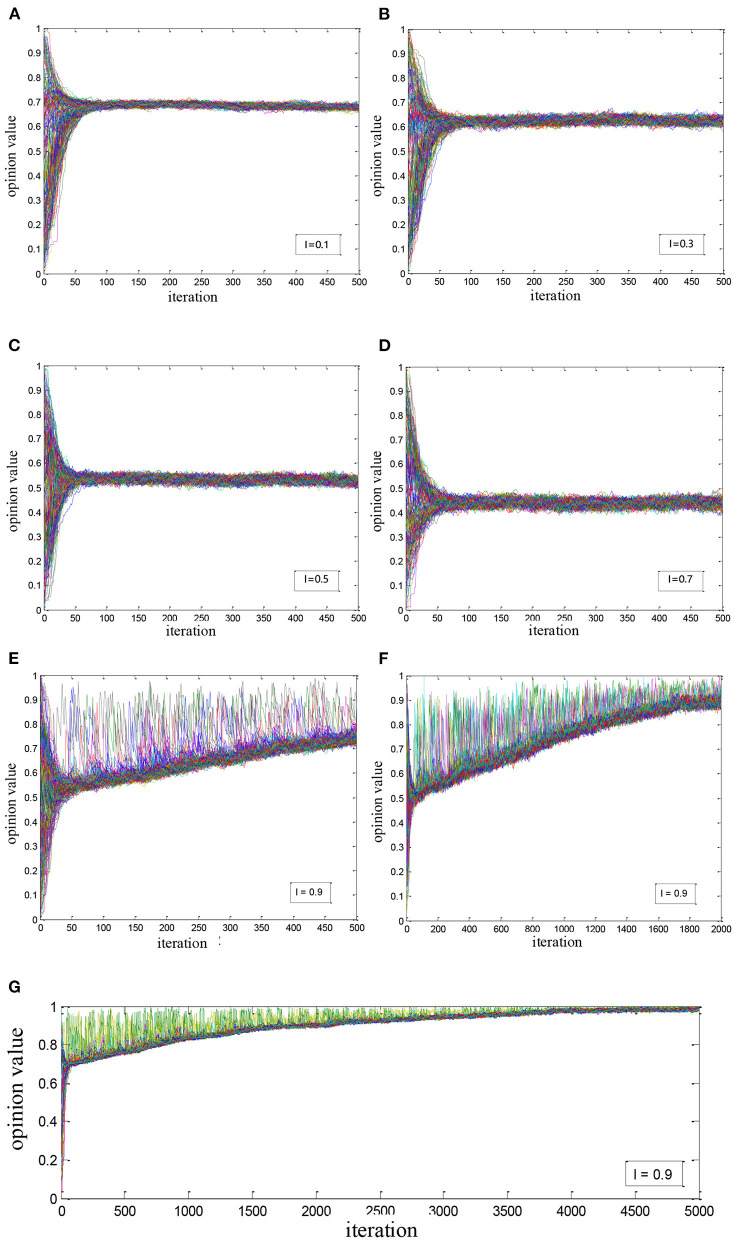
Simulation of health opinion evolution considering the network noise: **(A)** *I* = 0.1; **(B)** *I* = 0.3; **(C)** *I* = 0.5; **(D)** *I* = 0.7; **(E)** iteration = 500; **(F)** iteration = 2,000; **(G)** iteration = 5,000.

We introduced the network noise impact factor *I* based on the second experiment, and there were two cases:

(1) When there was noise and medical elites but no super-influencer engaged in the network, we supposed that the influence coefficient of medical elites *u*_*e*_ equaled the median of the change interval, i.e., equaled 0.5. We tried to study the evolutionary trend by debugging the noise intensity as shown in Figures 4A–D. It is found that:
  - The overall health opinion in the network no longer converges completely. And with the noise intensity increasing, health opinions among users fluctuate more and more intensely;
  - With the network noise increasing, the overall health opinion value of the network is becoming more negative gradually from the initial range of 0.65–0.7 to 0.4–0.5. When the level of noise reaches 0.7, the mid-value of the overall health opinion is close to 0.45 which is a negative opinion.
(2) When there was noise, a super-influencer but no medical elites engaged, an experiment was carried out to study the guidance of super-influencers on health opinions. As shown in Figures 4E–G, it is found that:
  - The super-influencer has a stronger influence on the overall health opinion evolution in the network. When the noise intensity reaches 0.9, the overall health opinion can still significantly converge to the opinion value of the super-influencer with enough iterations;
  - In the absence of medical elites, the performance of network noise interfering with the propagation of overall health opinion is obvious, opinions are diverse and it is difficult to reach a consensus in a short term.

#### Full Silence Model Simulation

**Figure 5:**
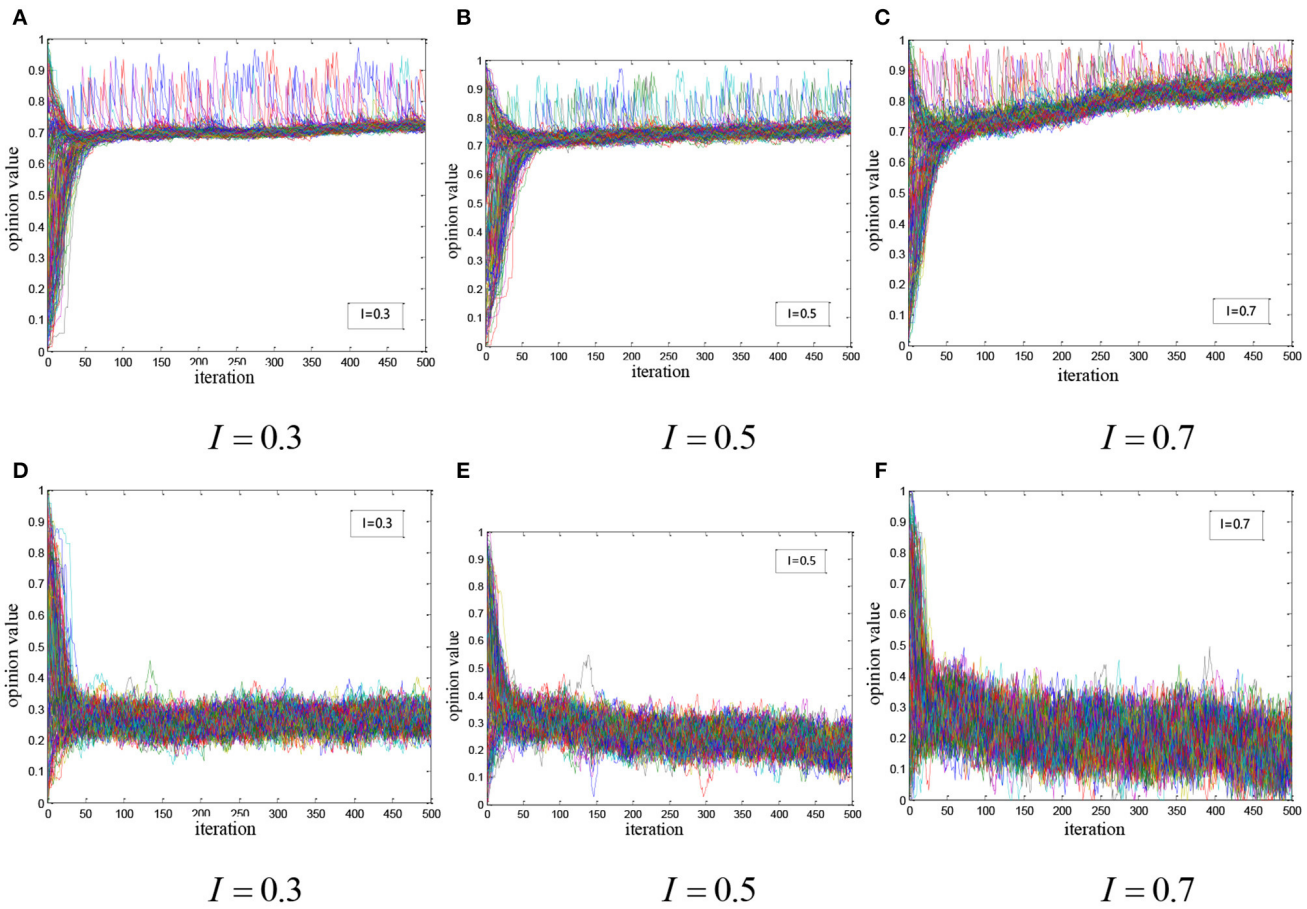
Simulation of health opinion evolution based on elite silence: **(A)** *I* = 0.3; **(B)** *I* = 0.5; **(C)** *I* = 0.7; **(D)** *I* = 0.3; **(E)** *I* = 0.5; **(F)** *I* = 0.7.

In this section, we considered the situation, in which ordinary users, medical elites, super-influencers, and network noise got involved together. There were two cases according to the health opinion attitude of super-influencer:

(1) The super-influencer held a positive health opinion. We studied the state of network opinion evolution by debugging the intensity of noise. As shown in Figures 5A–C, it is found that:
  - With the network noise increasing, the overall network opinion tends to fluctuate more violently so that it is more difficult to reach a consensus, while the fluctuation of the overall health opinion decreases instead;
  - The louder noise has a greater effect on the elite silence. When *I* = 0.7, although health opinions are not convergent, the overall opinion is significantly improved due to the elite silence.
(2) The super-influencer held a negative health opinion. We observed the evolution of network opinions by changing the intensity of the network noise, and the results are shown in Figures 5D–F. It is found that:
  - The super-influencer is more influential than medical elites, and the overall health opinion in the network approaches to a more negative health opinion after some iterations.
  - The network opinion is no longer convergent, all the health opinions are distributed on a larger interval, and the divergence is positively correlated with the noise level.
  - The negative opinion leader magnifies the effect of medical elite silence. When *I* = 0.5, the overall health opinion significantly approaches the opinion of the super-influencer.

In the comprehensive analysis, when the super-influencer holds a positive opinion, health information is less affected by the medical elite silence. When the noise level is 0.7, the health opinions of elites are significantly diminished. However, when the super-influencer holds a negative opinion, the influence of the health opinions of elites is significantly weakened when the noise level reaches 0.5.

## Conclusions

Health opinion dissemination in the network has become an important issue, which significantly affects public health. Based on noise-silence causality, this paper established an opinion evolutionary model of public health to study the medical elite silence and compare the influence of user type and silence on health opinion propagation through simulative experiments. The main findings in this study can be concluded as follow.

Network noise is an important factor that interferes with the dissemination of health opinions. Under the ideal network condition without elite users, super-influencers, and noise, the overall network opinion converged in a short time. In particular, the appearance of noise that represented network violence brought great uncertainty to the dissemination of health information. People's opinions on health presented great controversy and uncertainty, which may bring great hidden danger to the health choices of individuals and groups.

Super-influencers are at the heart of the exchange of health opinions. Although the number of super-influencers was fewer than medical elites, they had a greater impact on the health cognition of the whole network, which was fundamental. Even with a lot of noisy information in the network, super-influencers were not easily affected due to their position of super advantage. Furthermore, the group health opinion showed the interval fluctuation. With the increase of time, such fluctuation gradually reduced, and the opinion gradually approached the opinion of the super-influencers.

The silence effect of the medical elite showed different impacts. In the absence of super-influencers, the medical elite silence led to a small vibration of health opinion in the network group. With the increase of noise, the vibration amplitude also increased, but it is basically in a state of convergence. With the engagement of super-influencers with positive opinions, the effect of medical elite silence had limited influence on the communication of health opinions. When the noise reached a higher level, the silence effect had an obvious influence on the opinions. However, when super-influencers held negative opinions, the impact of the silence effect had become obvious if the noise reached a medium level.

There are three suggestions for institutions or government sectors who care about a good information environment for public health. Firstly, noise control, especially of noise effecting medical elites, who are vulnerable to be attacked on social networks in a disputed and fragmented society nowadays. Words with no support should not be encouraged, gatekeeping mechanisms in internet information release should be enhanced. Secondly, the medical elite should be encouraged to share points, and different points of view among them should be permitted, even welcomed, if supported by evidence. A tolerant environment for medical elites is urgently needed, even policy specifically to protect them could be considered. Thirdly, the attitudes of super-influencers are very important, a super-influencer can change public opinion to a large extent, so a super-influencer with a positive attitude is of vital importance, which is the key point.

## Data Availability Statement

The raw data supporting the conclusions of this article will be made available by the authors, without undue reservation.

## Author Contributions

JW and CQ: conceptualization, methodology, software, data curation, and writing-original draft preparation. HJ and LG: data curation and writing reviewing and editing. JC and YX: writing-original draft preparation and funding. All authors contributed to the article and approved the submitted version.

## Funding

This research was funded by the National Social Science Fund of China, grant number 18BTQ050.

## Conflict of Interest

The authors declare that the research was conducted in the absence of any commercial or financial relationships that could be construed as a potential conflict of interest. The reviewer JH declared a shared affiliation with several of the authors JW and CQ, to the handling editor at time of review.

## Publisher's Note

All claims expressed in this article are solely those of the authors and do not necessarily represent those of their affiliated organizations, or those of the publisher, the editors and the reviewers. Any product that may be evaluated in this article, or claim that may be made by its manufacturer, is not guaranteed or endorsed by the publisher.

## Footnotes

^1^Facebook, www.facebook.com; Twitter, twitter.com; Wechat, https://www.wechat.com/en.
